# Application of a Stability-Indicating HPTLC Method for Simultaneous Quantitative Determination of Olmesartan Medoxomil and Hydrochlorothiazide in Pharmaceutical Dosage Forms

**DOI:** 10.1155/2013/363741

**Published:** 2013-11-04

**Authors:** Kaliappan Ilango, Pushpangadhan S. Shiji Kumar

**Affiliations:** ^1^Department of Pharmaceutical Chemistry, SRM College of Pharmacy, SRM University, Kattankulathur, Kancheepuram, Tamil Nadu 603 203, India; ^2^Department of Pharmaceutical Chemistry, Jamia Salafiya Pharmacy College, Pulikkal, Malappuram, Kerala 673 637, India

## Abstract

A rapid, precise, sensitive, economical, and validated high performance thin layer chromatographic method is developed for simultaneous quantification of olmesartan medoxomil and hydrochlorothiazide in combined tablet dosage form. The method used amlodipine as internal standard (IS). Chromatographic separations were achieved on silica gel 60 F_254_ plates using toluene-methanol-ethyl acetate-acetone (2.5 : 1 : 0.5 : 2, v/v/v/v) as mobile phase. Densitometric analysis was carried out in the reflectance mode at 258 nm. Calibration curves were linear over a range of 80–480 ng/band for olmesartan medoxomil and 25–150 ng/band for hydrochlorothiazide. The detection and quantification limits were found to be 18.12 and 56.35 ng/band for olmesartan medoxomil and 6.31 and 18.56 ng/band for hydrochlorothiazide, respectively. Intra- and interassay precision provided relative standard deviations lower than 2% for both analytes. Recovery from 99.60 to 101.22% for olmesartan medoxomil and 98.30 to 99.32% for hydrochlorothiazide show good accuracy. Both the drugs were also subjected to acid, alkali, oxidation, heat, and photodegradation studies. The degradation products obtained were well resolved from pure drugs with significantly different *R*_*f*_ values. As the method could effectively separate the drugs from their degradation products, it can be used for stability-indicating analysis. Validation of the method was carried out as per international conference on harmonization (ICH) guidelines.

## 1. Introduction

**Figure 1:**
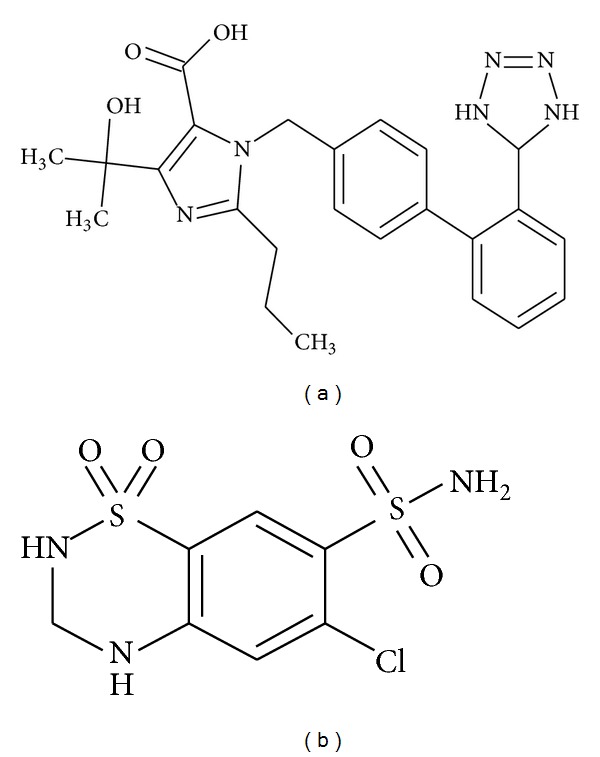
Chemical structure of (a) olmesartan medoxomil (b) hydrochlorothiazide.

Olmesartan medoxomil (OLM), chemically 2,3-dihydroxy-2-butenyl 4-(1-hydroxy-1-methylethyl)-2-propyl-1-[p-(*O*-1H-tetrazol-5-yl phenyl) benzyl] imidazole-5-carboxylate, cyclic 2,3-carbonate is a prodrug and it is hydrolysed to olmesartan during absorption from the gastrointestinal tract (Figure 1(a)). It is a selective AT1 subtype angiotensin II receptor antagonist. Hydrochlorothiazide (HTZ), chemically 6-chloro-3,4-dihydro-2,4-1,2,4-benzothiadiazine-7-sulfonamide-1,1-dioxide (Figure 1(b)), is a widely used thiazide diuretic [1–3]. Olmesartan and hydrochlorothiazide are available in the market as a combined dosage form for treatment of hypertension. Extensive literature survey revealed determination of OLM in dosage form by UV-visible spectrophotometry [4, 5], HPLC-UV [6, 7], and capillary electrophoresis [8]; in biological fluids by HPLC [9] and LC-MS [10, 11]. Determination methods of HTZ in pharmaceutical dosage form and biological fluids include chemiluminescence [12], HPLC [13], and electrochemical study [14]. Determination methods of OLM and HTZ combination include UV-spectrophotometry [15–18], RP-HPLC, and HPTLC [19, 20]. 

Till now, no stability indicating HPTLC method using an internal standard has been reported for simultaneous determination of OLM and HTZ for the combined dosage form. The objective of the present work is to develop and validate simple, sensitive, economic, rapid, precise, and accurate stability indicating HPTLC method for simultaneous determination of OLM and HTZ in accordance with ICH guidelines. HPTLC enables rapid analysis at lower cost than other techniques with selectivity, accuracy, and reproducibility. HPTLC also facilitates repeated detection (scanning) of chromatogram with the same or different parameters. In HPTLC, the consumption of mobile phase is quite low. Therefore the proposed method is very useful for routine analysis in quality control and can be used for assessing the stability of OLM and HTZ in pharmaceutical preparations.

## 2. Experimental

### 2.1. Chemicals, Reagents, and Solutions

Pharmaceutical grade olmesartan medoxomil (≥98.0%), hydrochlorothiazide (≥99.0%), and internal standard amlodipine IS (≥99.0%) were supplied by A To Z Laboratories, Chennai, India. Tablets, Olmesar-H (Macleods Pharmaceuticals Pvt. Ltd) and Olmy-H (Zydus Cadila Healthcare Ltd) both labeled to contain 40 mg OLM and 12.5 mg HTZ, were purchased from local pharmacy. Analytical grade methanol, toluene, ethyl acetate, and acetone (97 to 98% v/v) were all obtained from M/s. Merck Chemicals, Mumbai, India. Stock solutions (1.0 mg/mL) of compounds were prepared by dissolving 25 mg of OLM, IS, and HTZ standards individually into a 25 mL volumetric flask using methanol. A series of solutions containing mixture of drugs was prepared by transferring appropriate aliquots from standard stock solutions and diluting to volume with methanol. The final solution contained 8–48 *μ*g/mL OLM, 2.5–15 *μ*g/mL HTZ, and 5–30 *μ*g/mL IS. The concentration was fixed, taking into account the proportion in which OLM and HTZ are present in tablet formulation (3.2 : 1 ratio).

### 2.2. Chromatography

Chromatographic separation of drugs was performed on Merck TLC plates precoated with silica gel 60 F_254_ (10.0 × 10.0 cm with 250 mm layer thickness) from E. Merck, Germany. Time for chamber saturation was optimized to 10 minutes. Sample and standard zones were applied to the plates as bands (number of tracks 7, track distance from the left plate edges 20 mm, distance from plate bottom 10 mm, band length 1 mm, and distance between bands 10 mm) by means of Camag 100 *μ*L sample syringe (Hamilton, Switzerland) with a Linomat 5 applicator (Camag, Switzerland). The flow rate was 10 *μ*Ls^−1^ by using a nitrogen aspirator. The plates were left to equilibrate for 3 minutes in a 10.0 × 10.0 cm horizontal chamber (Camag, Switzerland) and then developed to a distance of 80 mm using toluene : methanol : ethyl acetate : acetone (2.5 : 1 : 0.5 : 2, v/v/v/v) as mobile phase. Separation was obtained within 10 minutes, and before detection, the plates were dried at 60°C for 4 minutes to eliminate mobile phase. Densitometric scanning was performed in the absorbance/reflectance mode at 258 nm, using Camag TLC scanner 3 with deuterium source, slit dimension settings of length 2 mm, width 0.1 mm, monochromator band width 30 nm, and scan rate of 4 mms^−1^. WinCATS software (V1.4.2, Camag, Switzerland) was used for scanner control and data processing. Electronic balance (Shimadzu model AY-120, Japan) was used for weighing purposes. The optimum absorption wavelength (258 nm) was determined by measuring in situ UV absorption spectrum of standard OLM and HTZ. The whole procedure took no more than 20 minutes. The *R*_*f*_ values of OLM, HTZ, and IS were found to be 0.29 ± 0.02, 0.52 ± 0.01, and 0.71 ± 0.01, respectively. Concentrations of the compounds chromatographed were determined from the diffusely reflected light. 

### 2.3. Method Validation

The proposed HPTLC method was validated for selectivity, precision, accuracy, linearity, robustness, LOD, LOQ, and recovery according to ICH guidelines [21, 22].

### 2.4. Selectivity

The selectivity of the method was assessed by analyzing in triplicate the standard and sample. The bands of OLM and HTZ from pharmaceutical formulation were confirmed by comparing *R*_*f*_ values with those from standard. The peak purity was determined by win CATS software. The purity of each compound was confirmed by analyzing UV spectrum at start, apex, and end of peak. 

### 2.5. Linearity and Sensitivity

For preparation of calibration plot, 10 *μ*L each of mixed standard solutions containing 8–48 *μ*g/mL OLM, 2.5–15.0 *μ*g/mL HTZ, and 5–30 *μ*g/mL IS were applied to the plate to furnish concentrations of 80–480 ng/band OLM, 25–150 ng/band HTZ, and 50–300 ng/band IS, respectively. Each standard was analysed in triplicate and peak areas were recorded. Calibration plots were constructed separately by plotting mean RRF (relative retention factor, peak area ratio of OLM or HTZ to IS) against respective concentrations of OLM and HTZ. The data were best fitted by linear equation *mx* + *b* = *y*. The sensitivity of the method was determined with respect to LOD, LOQ, linearity range, and correlation coefficient. The LOD and LOQ parameters were determined from the regression equations of OLM and HTZ. LOD = 3.3 × SD/*s*  and LOQ = 10 × SD/*s*, where SD is the standard deviation of the response and “*s*” is the slope of corresponding calibration curve. 

### 2.6. Precision

Precision is the measure of how the data values are close to each other for a number of measurements under the same analytical conditions and is expressed as relative standard deviation (%RSD). The precision study was carried at three different concentrations (low, medium, and high) of OLM (80, 320, and 480 ng/band) and HTZ (25, 100, and 150 ng/band) respectively. 

### 2.7. Accuracy

Accuracy is the measure of how the experimental value is close to the true value and is expressed as %RSD, standard error mean (SEM), and % recovery. Recovery studies by standard addition method were performed in a view to justify the accuracy of the proposed method. Previously analyzed samples containing OLM (80, 160, and 240 ng/band) and HTZ (25, 50, and 75 ng/band) were spiked with standard OLM (25, 50, and 75 ng) and standard HTZ (25, 50, and 75 ng). The mixtures were analyzed in triplicate by the proposed method. 

### 2.8. Robustness

The robustness of an analytical procedure refers to its ability to remain unaffected by small and deliberate variations in method parameters and provides an indication of its reliability for routine analysis. To determine the robustness of method, the experimental conditions were deliberately altered and retention factor (*R*_*f*_), assay percent, and %RSD were evaluated. Conditions altered were mobile phase composition, development distance, time of spotting to chromatography, and detection wavelength. For all changes in conditions, the sample was analyzed in triplicate. When the effect altering one set of conditions was tested, the other conditions were held constant at optimum values.

### 2.9. Solution Stability

The stability of the drugs in solution during analysis was determined by repeated analysis of samples during the course of experimentation on the same day and also after storage of drug solution for five days under laboratory conditions (25 ± 2°C) and under refrigeration (2–8°C). The samples were analyzed immediately and after a period of one, three, and five days. 

### 2.10. Forced Degradation Studies

Forced degradation studies are performed to unequivocally identify the analyte of interest among other compounds expected to be present.

#### 2.10.1. Acid-Induced Degradation Study

In this study, hydrochloric acid (0.1 M, 5 mL) was added to 25 mL volumetric flask containing 0.8 mL, 0.25 mL, and 0.5 mL of methanolic stock solutions of OLM, HTZ, and IS, respectively. The mixtures were refluxed at 50°C for 1 hour and completed to volume with methanol. 10 *μ*L of resulting solution was spotted as bands to furnish concentration of 320 ng/band OLM, 100 ng/band HTZ, and 200 ng/band IS, respectively. The procedure was performed in dark excluding possible degradation from the effect of light. 

#### 2.10.2. Base-Induced Degradation Study

Sodium hydroxide (0.1 M, 5 mL) was added to 25 mL volumetric flask containing 0.8 mL, 0.25 mL, and 0.5 mL of methanolic stock solutions of OLM, HTZ, and IS, respectively. The mixtures were refluxed at 50°C for 1 hour and completed to volume with methanol. 10 *μ*L of resulting solution was spotted as bands to furnish concentration of 320 ng/band OLM, 100 ng/band HTZ, and 200 ng/band IS, respectively. The procedure was performed in dark excluding possible degradation from the effect of light.

#### 2.10.3. Hydrogen Peroxide-Induced Degradation Study (Oxidation)

Hydrogen peroxide (3.0%, 5 mL) was added to 25 mL volumetric flask containing 0.8 mL, 0.25 mL, and 0.5 mL of methanolic stock solutions of OLM, HTZ, and IS, respectively. The mixtures were refluxed at 50°C for 1 hour and completed to volume with methanol. 10 *μ*L of resulting solution was spotted as bands to furnish concentration of 320 ng/band OLM, 100 ng/band HTZ, and 200 ng/band IS, respectively. The procedure was performed in dark excluding possible degradation from the effect of light.

#### 2.10.4. Wet Degradation Study

For wet degradation study, 0.8 mL, 0.25 mL, and 0.5 mL of methanolic stock solutions of OLM, HTZ, and IS were transferred to 25 mL volumetric flask. To this, 5.0 mL methanol was added and the sample was refluxed at 60°C for 1 hour. The volume was made up with methanol and 10 *μ*L was applied to plate and analyzed under optimized chromatographic conditions. 

#### 2.10.5. Photodegradation Study

For photodegradation study, solution containing 320 ng/band OLM, 100 ng/band HTZ, and 200 ng/band IS was exposed to UV light (365 nm) in a photostability chamber for 24 hours. The resulting solution was analyzed under optimized chromatographic conditions.

### 2.11. Analysis of Marketed Tablet Dosage Form

The application of the developed method was evaluated to determine the amounts of OLM and HTZ in their marketed tablet dosage form. Twenty tablets, each of both brands (Olmesar-H and Olmy-H), were accurately weighed. Their average weight was determined and pulverized to fine powder. A quantity of tablet powder equivalent to 40 mg OLM and 12.5 mg HTZ was accurately weighed and transferred to 100 mL volumetric flasks. The volume was adjusted with methanol and the resultant solution was sonicated for 15 minutes and filtered through 0.45 *μ*m nylon filters (Millipore, Milford, USA). From the resulting sample solution, 2 mL was transferred to 50 mL volumetric flask. To this 0.5 mL of standard stock solution (1.0 mg/mL) of IS was added and diluted to 50 mL with methanol to furnish a solution containing 16 *μ*g/mL OLM, 5 *μ*g/mL HTZ, and 10 *μ*g/mL IS, respectively. From the final solution 10 *μ*L was spotted as bands and analyzed. Quantification was done using relative retention factor (RRF) for OLM and HTZ. The procedure was repeated five times for analysis of homogenous samples. 

## 3. Results and Discussion

### 3.1. Chromatographic Method Development and Optimization

**Figure 2:**
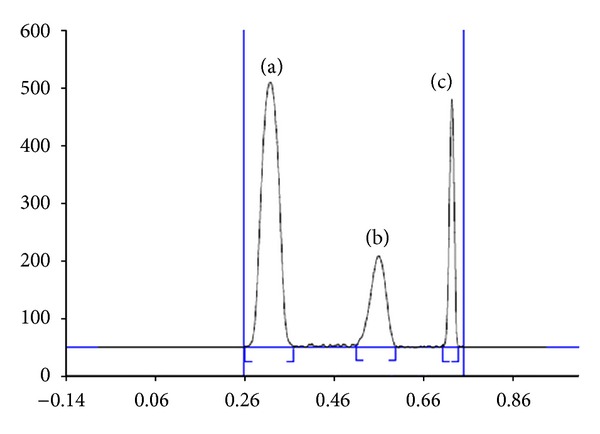
Representative chromatogram of OLM (*R*_*f*_  0.29), HTZ (*R*_*f*_  0.52), and IS (*R*_*f*_  0.71).

**Figure 3:**
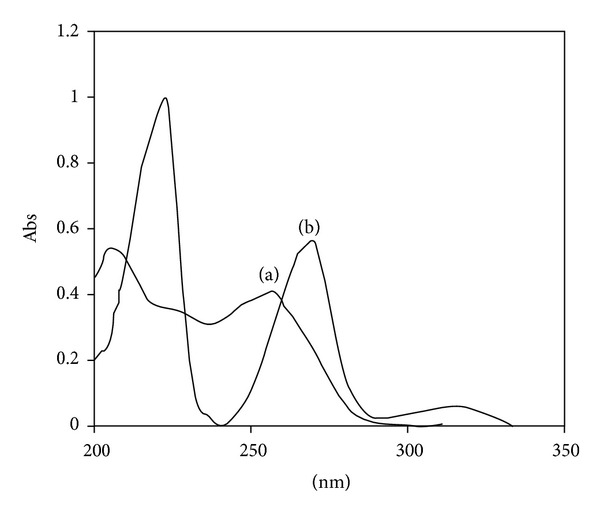
Typical overlaid absorption spectra of (a) OLM and (b) HTZ.

Preliminary experiments were carried out to optimise the parameters affecting simultaneous estimation of both drugs using HPTLC and detection at 258 nm. The solvent type, solvent ratio, and detection wavelength were varied to determine the chromatographic conditions giving best separation. Different solvent types like methanol : ethyl acetate : acetic acid (4 : 2.5 : 0.5, v/v/v), toluene : methanol : ethyl acetate : acetone (2.5 : 1 : 0.5 : 2, v/v/v/v), and toluene : ethyl acetate : acetic acid (7 : 2 : 0.5, v/v/v) were investigated for complete chromatographic resolution of two drugs. Mobile phase consisting of toluene : methanol : ethyl acetate : acetone (2.5 : 1 : 0.5 : 2, v/v/v/v) was found to give the best sensitivity, efficiency, and peak shape. It gave symmetric, well-resolved spots with *R*_*f*_ values of OLM 0.29 ± 0.02, HTZ 0.52 ± 0.01, and IS 0.71 ± 0.01 as shown in Figure 2. The UV spectra of all three analytes were determined independently and in combination. It was observed from overlain spectra (Figure 3) that at wavelength 258 nm, all three drugs could be detected simultaneously with no mobile phase interference, good separation, sensitivity, and consistent baseline. All experiments were performed at 28°C temperature.

### 3.2. Method Validation

The method was validated for parameters such as linearity, specificity, LOD, LOQ, precision, accuracy, and robustness. 

**Figure 4:**
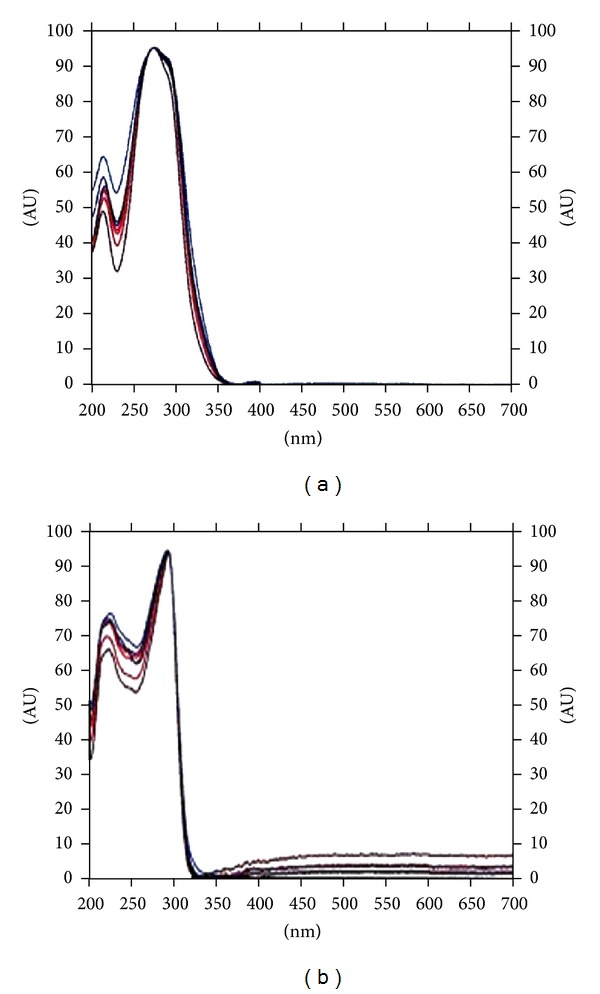
Peak purity spectra of (a) OLM and (b) HTZ. The wavelength selected for analysis was 258 nm.

Densitogram obtained shows no interference between peaks or from other constituents originating from the excipients of tablets. It can also be assumed from peak purity spectra (Figure 4) that the method is specific for these analytes, which enables reliable results to be obtained. The peak purity of more than 0.99 indicates no interference from any impurities in the separation and determination of OLM and HTZ peaks.

The calibration plots were linear in the concentration range 80–480 ng/spot (*n* = 3, *r* = 0.9996) for OLM and 25–150 ng/spot (*n* = 3, *r* = 0.9994) for HTZ, respectively. The low values of standard deviation showed that the standard error of slope and the intercept of ordinate showed that the calibration plot did not deviate from linearity.  Table 1 shows the linearity parameters of calibration curve.

The intra- and interday precision values were calculated for three concentrations of OLM and HTZ (Table 2). The RSD values were 0.13–0.99 and 0.38–1.73 for intra- and interday, respectively. The low RSD values (<2%) indicate sensitivity and repeatability of the proposed method. Reproducibility checked by different analyst shows no significant difference (RSD ≤ 1.5%) in the intra- and interday precision.

The recovery study performed at three different concentrations in triplicate shows good recoveries, 99.60–101.22% for OLM and 98.30–99.32% for HTZ, respectively. The % RSD and % relative error in all cases were within the acceptable limit (<2%). The results of recovery study are reported in Table 3.

Results of robustness study are depicted in Table 4. The retention factor (*R*_*f*_) and assay (%) were not significantly affected. RSD (%) values in all robustness parameters were examined and found to be within the limit of <2%. The result showed that proposed method is robust and was acceptable for proving data of acceptable quality.

**Figure 5:**
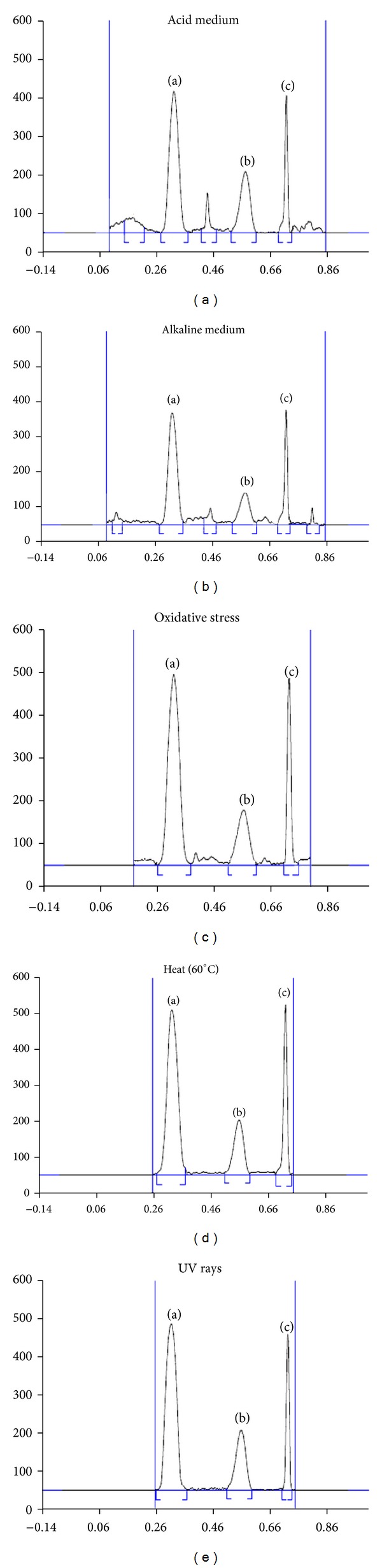
Densitogram obtained after stress testing under different conditions.

 The results of specificity studies showed that OLM and HTZ are stable when exposed to different stress conditions. No significant deviation of *R*_*f*_ values or variations of assay values were observed. Densitograms obtained from samples stressed under different conditions are given in Figure 5.  Table 5 shows percentage degradation of OLM and HTZ peaks.

The validated HPTLC method was applied for simultaneous determination of OLM and HTZ in commercial tablets (Olmesar-H and Olmy-H). For both brands, 10 *μ*L of final sample solution containing 160 ng/band OLM, 50 ng/band HTZ, and 100 ng/band IS was spotted. The chromatogram obtained is identical to that of drug standard solution, without any interference from excipients. The results as depicted in Table 6 indicate that each drug in tablet corresponds to requirements of label claim. The low RSD values (<2%) confirmed the suitability of method for routine analysis of OLM and HTZ in pharmaceutical dosage forms. Also the proposed method was compared with the reported HPLC method (Table 7).

## 4. Conclusion

The developed HPTLC method combined with densitometry was found suitable for determination of OLM and HTZ in bulk and tablet dosage form without any interference from excipients. Statistical analysis proves that the developed method is repeatable, reproducible, and selective for the analysis of both drugs. Its advantages are low cost of reagents (only 10 *μ*L of solution is applied on plate), speed (the entire process took no more than 20 minutes), sensitivity, accuracy and precision. The method has lower LOD and LOQ values and high recovery.

## Figures and Tables

**Table 1:** Linearity parameters for calibration curve.

Parameter	Olmesartan medoxomil	Hydrochlorothiazide
Retention factor (*R*_*f*_)	0.29	0.52
Linearity range (ng/band)	80–480	25–150
Regression equation (*y* = *mx* + *b*)	*y* = 1.32*x* + 0.95	*y* = 3.54*x* + 1.02
Correlation coefficient (*r*)	0.9996	0.9999
Limit of detection (ng/band)	18.12	6.31
Limit of quantification (ng/band)	56.35	18.56
Standard deviation of slope^a^ (*S*_*a*_)	0.9813	0.9012
Standard deviation of intercept^a^ (*S*_*b*_)	1.2013	1.1430
Regression coefficient (*r* ^2^)	0.999	0.998
Peak purity index (*P*)	0.9986	0.9995
Method precision^a^ (RSD %)	0.92	0.84

^a^Mean of five replicates, RSD: relative standard deviation.

**Table 2:** Results of precision studies of proposed method.

Drug concentration (ng/band)	Intraday precision		Interday precision	
	Calculated amount^a^ ± SEM (ng/band)	% RSD	Calculated amount^a^ ± SEM (ng/band)	% RSD
OLM				
80	79.12 ± 0.20	0.44	82.41 ± 0.47	0.98
320	323.10 ± 0.24	0.13	325.60 ± 0.90	0.48
480	485.62 ± 0.94	0.34	483.13 ± 1.36	0.38
HTZ				
25	26.15 ± 0.15	0.99	25.93 ± 0.26	1.73
100	102.36 ± 0.29	0.50	101.95 ± 0.64	1.08
150	156.91 ± 0.71	0.78	153.12 ± 1.53	1.08

^a^Mean of three replicates, RSD: relative standard deviation, SEM: standard error mean.

**Table 3:** Accuracy study by standard addition method.

Drug	Initial amount (ng/band)	Fortified amount (ng/band)	Amount recovered^a^ ± SEM (ng/band)	Mean recovery (%)	% RSD
Olmesartan medoxomil	80	25	105.92 ± 0.24	100.88	0.40
	160	50	212.56 ± 0.50	101.22	0.50
	240	75	313.71 ± 1.22	99.60	0.37

Hydrochlorothiazide	25	25	49.15 ± 0.14	98.30	0.51
	50	50	99.32 ± 0.31	99.32	0.54
	75	75	148.53 ± 0.55	99.02	0.64

^a^Mean of three replicates, RSD: relative standard deviation, SEM: standard error mean.

**Table 4:** Results from robustness study.

Condition	Retention factor (*R*_*f*_)		Assay^a^ (%)		% RSD	
	OLM	HTZ	OLM	HTZ	OLM	HTZ
Mobile phase composition (v/v/v/v)						
Toluene : methanol : ethyl acetate : acetone						
(2.4 : 1 : 1 : 1.5)	0.31	0.54	100.70	101.20	0.41	0.69
(2.5 : 1 : 1 : 2.5)	0.32	0.53	99.30	100.76	1.21	1.08
(2.5 : 0.9 : 0.9 : 1.5)	0.31	0.54	98.72	97.91	0.93	0.86
Development distance (cm)						
6	0.30	0.54	98.76	101.21	0.71	0.19
7	0.31	0.52	97.86	99.30	0.27	0.87
Time of spotting to chromatogram (min)						
9	0.30	0.52	98.61	99.32	1.32	1.47
10	0.28	0.52	100.86	101.20	1.62	0.72
Detection wavelength (nm)						
275	0.29	0.53	98.71	99.41	1.01	0.43
280	0.29	0.54	98.76	99.37	0.37	0.86

^a^Mean of three determinations, RSD: relative standard deviation.

**Table 5:** Results from degradation study.

Degradation condition	% Degradation	Number of degradation products (*R*_*f*_)
Acid	5%, 3%, 8%	0.015, 0.43
Alkali	12%, 18%, 5%	0.10, 0.45, 0.79
Oxidative	22%, 5%, 1%	0.20
Heat	—	—
Photolytic (UV)	—	—

**Table 6:** Results from HPTLC quantification of olmesartan medoxomil and hydrochlorothiazide in tablets.

Sample	Label claim (mg)		Amount present^a^ (mg)		SD		% RSD	
	OLM	HTZ	OLM	HTZ	OLM	HTZ	OLM	HTZ
Olmesar-H	40	12.5	41.05	12.42	0.17	0.21	0.41	1.69
Olmy-H	40	12.5	41.38	12.35	0.41	0.14	0.99	1.13

^a^Mean of five determinations, SD: standard deviation, RSD: relative standard deviation.

**Table 7:** Comparison of the proposed method with the reported HPLC method.

Parameter	HPLC		HPTLC	
	OLM	HTZ	OLM	HTZ
Linearity	4–24 *μ*g/mL	2.5–15 *μ*g/mL	80–480 ng/band	25–150 ng/band
LOD	0.44 *μ*g/mL	0.21 *μ*g/mL	18.12 ng/band	6.31 ng/band
LOQ	1.32 *μ*g/mL	0.63 *μ*g/mL	56.35 ng/band	18.56 ng/band
Tablet analysis (% recovery)	100.24	100.10	103.03	99.08
